# Locally Advanced Basal Cell Carcinoma With Orbital Invasion: Treatment With Neoadjuvant Hedgehog Pathway Inhibition and Palliative Enucleation

**DOI:** 10.7759/cureus.104131

**Published:** 2026-02-23

**Authors:** Matthew A Murphy

**Affiliations:** 1 Department of Supportive Care Medicine, Moffitt Cancer Center, Tampa, USA

**Keywords:** basal cell carcinoma, cutaneous oncology, enucleation, hedgehog pathway inhibitor, locally advanced basal cell carcinoma, ocular oncology, periocular basal cell carcinoma, vismodegib

## Abstract

A 63‑year‑old man with a history of heavy tobacco use presented to the hospital with a 2 × 3 cm necrotic lesion of the left temporal region associated with pain and progressive vision loss in the left eye. Biopsy of the left lateral orbital wall demonstrated locally invasive basal cell carcinoma (BCC). The patient was referred to a National Cancer Institute-designated comprehensive cancer center for further management. He was initiated on neoadjuvant hedgehog pathway inhibition with vismodegib and had an excellent oncologic response. Despite this, visual acuity in the left eye continued to decline and was accompanied by worsening proptosis and pain, which was refractory to multimodal analgesia. The patient was referred to ocular oncology, and vismodegib was discontinued after six months of treatment in anticipation of surgery. The patient underwent left eye enucleation and reported complete resolution of pain. He showed no evidence of residual disease four months after completion of vismodegib therapy. This report highlights a rare case of locally advanced periocular BCC with orbital invasion managed with neoadjuvant hedgehog pathway inhibition and palliative enucleation.

## Introduction

Basal cell carcinoma (BCC) is the most frequently occurring cancer worldwide [1], with the majority of cases arising in sun-exposed areas of the head and neck. Most BCCs follow an indolent course and are asymptomatic, as progression to locally advanced disease with invasion of surrounding tissues is uncommon. For example, periocular BCC with orbital invasion has an estimated incidence of approximately 2% [2]. When orbital invasion does occur, patients may experience a substantial symptom burden, including pain, proptosis, and progressive visual impairment, with significant implications for functional status and quality of life. Orbital extension typically occurs through mechanisms such as perineural spread and progressive bony erosion, pathways associated with more aggressive tumor biology and poorer prognosis compared with disease confined to the eyelid [2].

Periorbital BCC poses diagnostic challenges due to the complex anatomy of the orbit and the potential for deep tissue invasion, bony involvement, and perineural spread. Imaging plays a critical role in defining disease extent, particularly in cases with suspected orbital or neuro-ophthalmic involvement. While early-stage BCC is typically managed in the outpatient setting with margin-controlled excision performed by dermatologists, locally advanced BCC (laBCC) involving the orbit requires coordinated multidisciplinary care, including dermatology, medical oncology, ocular oncology, radiation oncology, and palliative care, to optimize both oncologic and functional outcomes [3].

laBCC represents a small but clinically significant subset of BCC, characterized by deep tissue invasion, large tumor size, recurrence after prior therapy, or involvement of critical anatomic structures that preclude straightforward surgical management. Surgical excision, including Mohs micrographic surgery, remains the first-line treatment for laBCC when complete resection can be achieved with acceptable functional and cosmetic outcomes [4]. In cases where surgery is contraindicated or would result in unacceptable morbidity, radiation therapy is an established alternative first-line modality [3], particularly for tumors involving the head and neck.

The pathogenesis of BCC is driven largely by abnormal activation of the hedgehog signaling pathway, a developmental pathway that is normally inactive in adult tissues [5]. Most tumors harbor mutations in PTCH1 or SMO, resulting in continuous downstream signaling and unchecked cellular proliferation. Clinically available hedgehog pathway inhibitors (HHIs) act by blocking SMO, the key signaling protein in this pathway, thereby suppressing the proliferative program on which BCC cells depend. This mechanism makes SMO inhibition a rational and effective targeted therapy for patients with locally advanced disease who are not candidates for surgery or radiation [5], or as neoadjuvant therapy to reduce tumor burden prior to definitive surgical intervention [6].

The use of systemic HHIs is an established treatment for laBCC, and many patients experience excellent oncologic responses [5,6]. However, clinical response to systemic therapy does not uniformly translate into improvement of local disease-related complications, particularly in periorbital BCC with orbital invasion, where persistent pain, vision loss, and functional impairment can profoundly diminish quality of life. We present this case to highlight this discordance and to emphasize the importance of a multidisciplinary approach in the management of orbital laBCC, which may include adjuvant palliative surgical interventions such as enucleation.

## Case presentation

**Figure 1:**
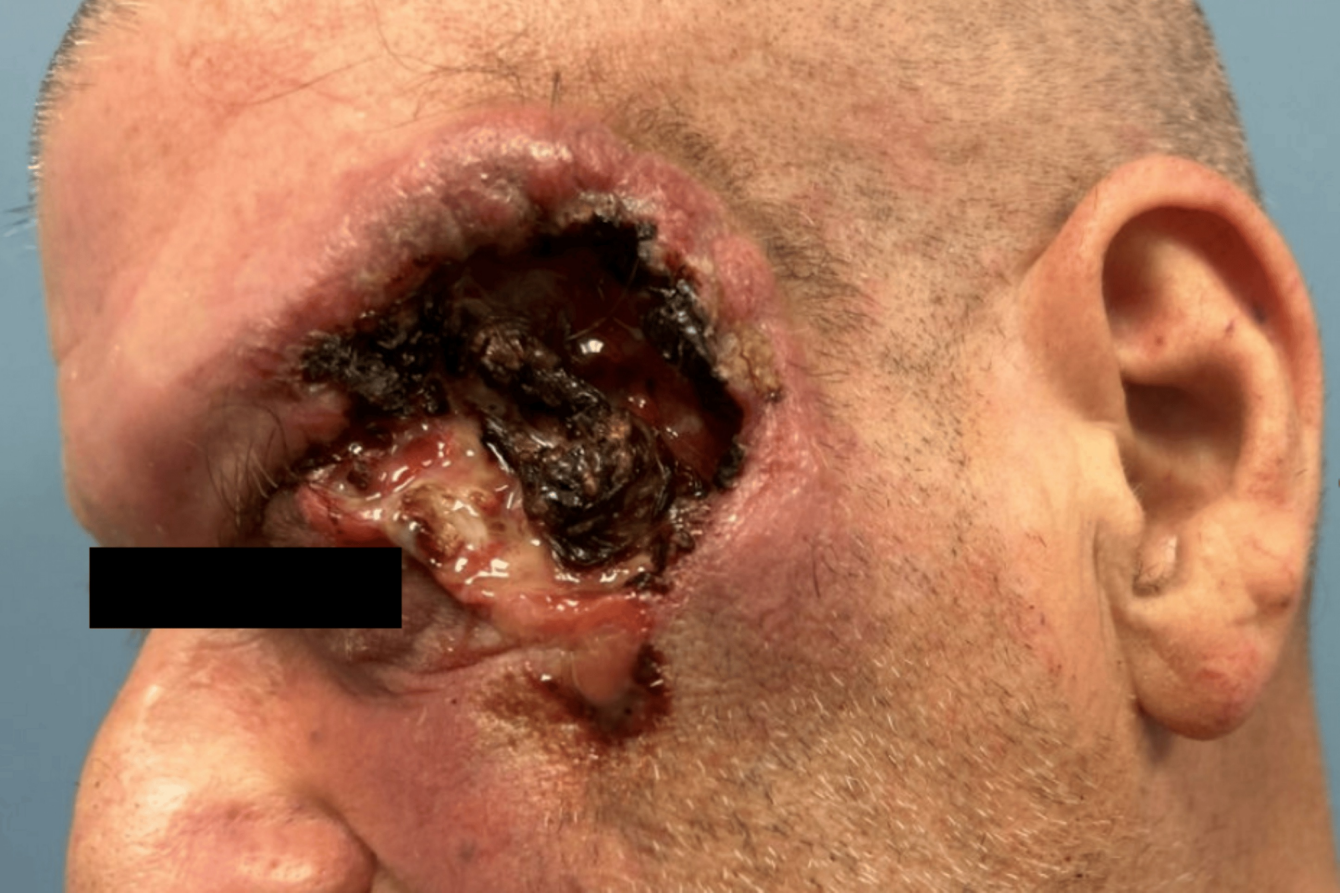
Left facial view demonstrating a necrotic lesion of the left temporal region with erosion into the left orbit.

A 63-year-old man with a history of heavy tobacco use presented to the hospital with a 2 × 3 cm necrotic lesion in the left temporal region, associated with pain and blurry vision in the left eye. Six months prior to presentation, the patient had noticed a small lesion on the left temple that gradually increased in size. He reported progressive pain and visual changes over several weeks before seeking medical attention. Physical examination revealed extension of the lesion into the left orbit (Figure 1). Computed tomography of the face demonstrated erosion of the left zygomatic bone, concerning for osteomyelitis, along with soft-tissue thickening, emphysema, and enhancement of the left paraseptal region extending into the lateral and superior extraconal orbital tissues, raising concern for early orbital cellulitis. Intravenous antibiotics were initiated. Biopsy of the left lateral orbital wall revealed basal cell carcinoma with nodular and infiltrative growth patterns.

**Figure 2:**
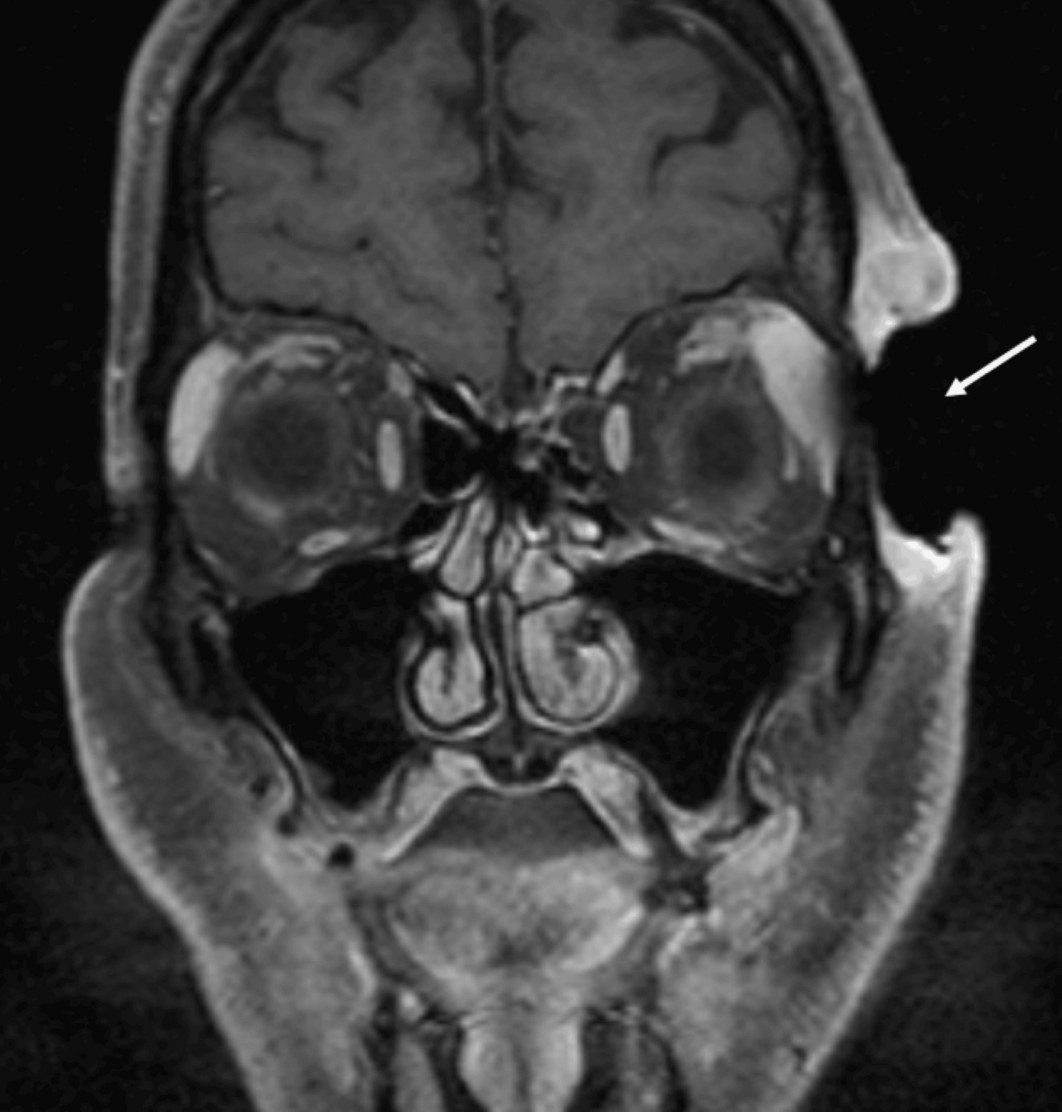
Coronal T1‑weighted post‑contrast fat‑suppressed MRI of the orbits demonstrating erosion of the left craniofacial soft tissues.

Ophthalmology was consulted during the patient’s hospitalization and recommended magnetic resonance imaging (MRI) of the orbits due to concern for orbital invasion. MRI revealed a 5-cm mass involving the left craniofacial soft tissues with orbital extension and lateral orbital bony erosion (Figure 2). The findings were highly suggestive of neoplastic involvement, with evidence of perineural spread along the ophthalmic (V1) branch of the trigeminal nerve at the supraorbital notch and into the superior extraconal orbit. The patient was subsequently referred to a National Cancer Institute-designated comprehensive cancer center for multidisciplinary evaluation and further management.

**Figure 3:**
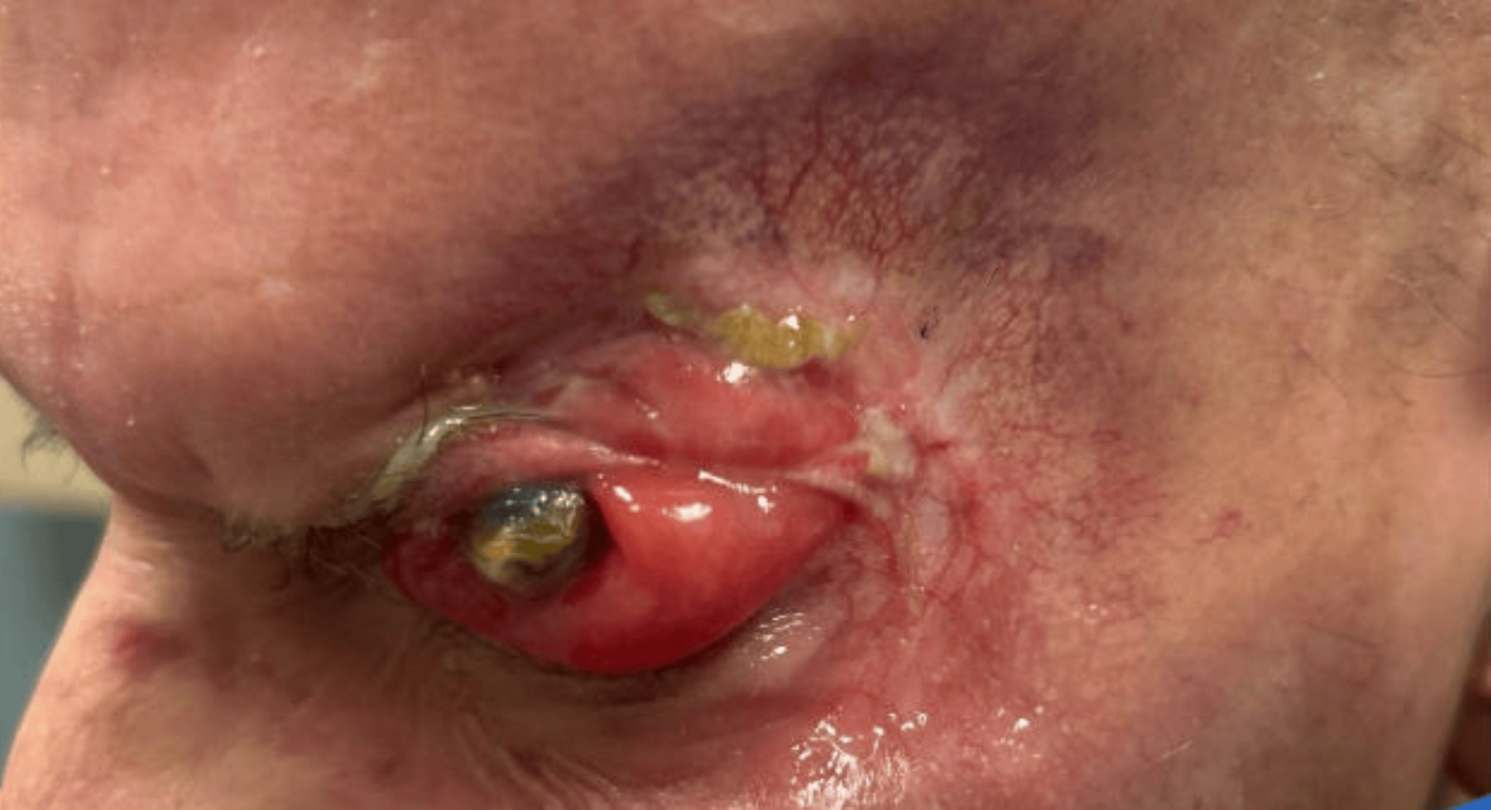
Left facial view demonstrating worsening proptosis of the left eye despite interval oncologic response after six months of treatment with hedgehog pathway inhibitor therapy.

The patient was initiated on neoadjuvant hedgehog pathway inhibition with vismodegib and evaluated by cutaneous oncology, radiation oncology, neuro-oncology, palliative medicine, and head and neck surgery. Following multidisciplinary discussion, continued treatment with vismodegib and definitive management with surgical resection were recommended. The patient expressed a preference to continue medical therapy to avoid surgical intervention, including enucleation. Although he demonstrated a positive oncologic response to vismodegib, visual acuity in the left eye declined to light perception only, accompanied by progressive proptosis (Figure 3) and worsening ocular pain that was refractory to multimodal analgesia. The patient was subsequently referred to ocular oncology by the palliative care team, and vismodegib was discontinued after six months of treatment in anticipation of planned surgical intervention.

**Figure 4:**
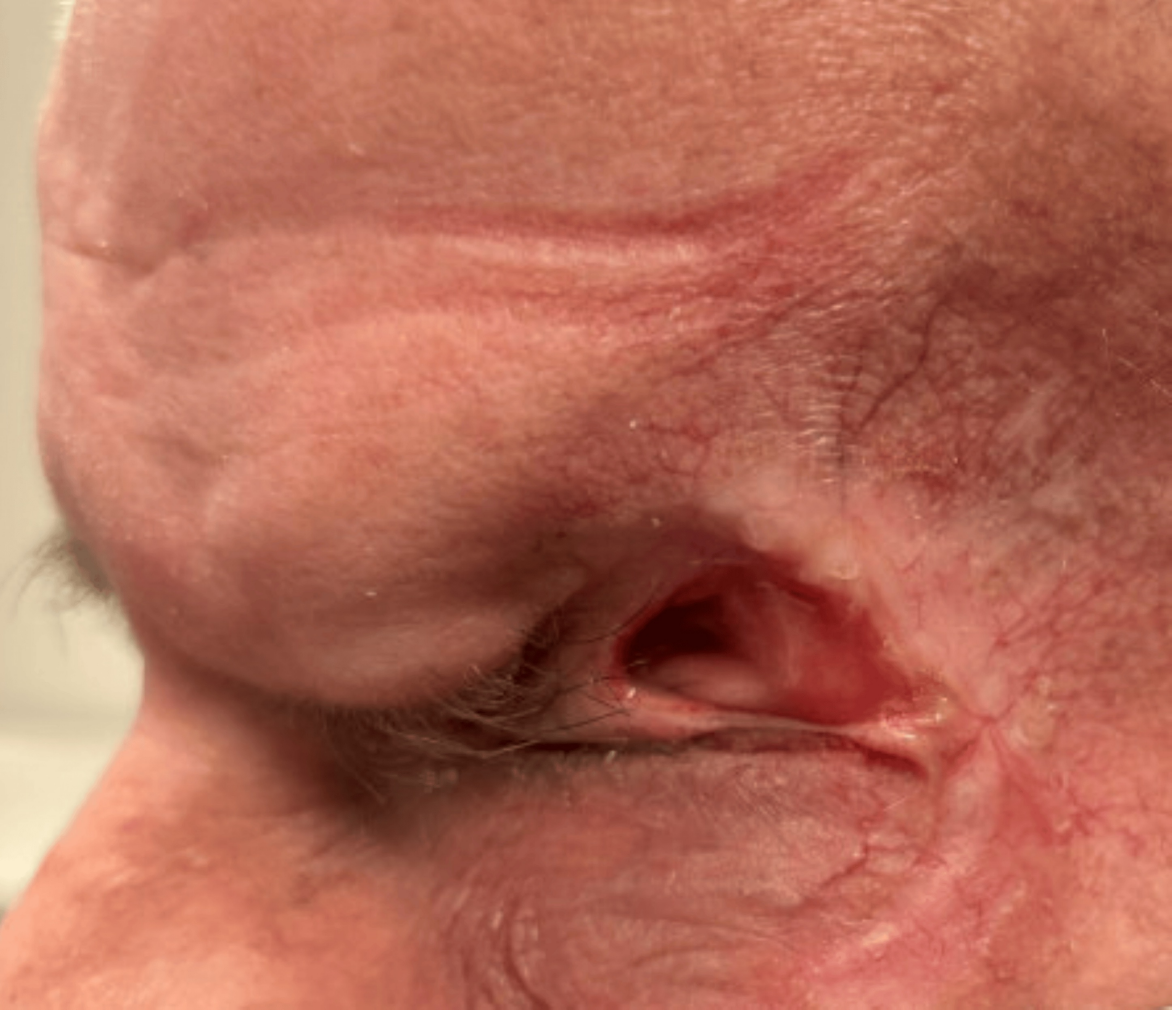
Left facial view following enucleation demonstrating no visible evidence of residual periocular disease four months after completing hedgehog pathway inhibitor therapy.

Ocular oncology recommended enucleation of the left eye for palliation of symptoms. The patient subsequently underwent enucleation and reported complete resolution of pain, enabling successful discontinuation of opioid analgesics and a marked improvement in patient-reported quality of life, despite resultant monocular vision. At the subsequent oncology follow-up, physical examination revealed no residual disease, and ongoing surveillance was recommended. Four months after completing treatment with vismodegib, the patient continued to show no evidence of recurrence on physical examination (Figure 4).

## Discussion

laBCC with orbital extension is uncommon but can result in significant morbidity due to local tissue destruction, bony invasion, and perineural spread [7,8]. When present, orbital involvement poses substantial diagnostic and therapeutic challenges and often necessitates a multidisciplinary approach to optimize both oncologic and patient-centered outcomes [3,7]. Collaboration among dermatology, medical and surgical oncology, ophthalmology, radiation oncology, and palliative care is frequently required, given the potential for significant symptom burden, including pain and visual impairment, which can profoundly affect functional status and quality of life [9].

The development of systemic HHIs has significantly expanded treatment options for patients with laBCC who are not surgical candidates or for whom surgical intervention would result in unacceptable morbidity [10,11]. Vismodegib has demonstrated meaningful clinical activity in patients with locally advanced disease, including those with periorbital involvement [11,12]. In the present case, however, despite a favorable oncologic response, the patient experienced progressive vision loss and worsening ocular pain. This discordance highlights an important clinical consideration: tumor regression does not necessarily translate into preservation of function, symptom relief, or improved quality of life, particularly in advanced periorbital disease where irreversible local or neuro-ophthalmic damage may already be present [9].

This case further illustrates the role of palliative enucleation in the management of laBCC involving the orbit. Although the patient initially hoped to avoid enucleation, persistent refractory pain and progressive visual decline prompted reconsideration following multidisciplinary evaluation and involvement of the palliative care team. Palliative enucleation has been shown to be effective in relieving intractable ocular pain when vision cannot be salvaged, and its role should be considered within a broader symptom-directed treatment framework [13]. Framing enucleation as both a palliative and quality-of-life-preserving intervention may help normalize its role in selected patients with refractory symptoms, even in the setting of effective cancer-directed therapy.

Although this patient demonstrated no clinical evidence of residual BCC four months after completion of vismodegib therapy, ongoing surveillance remains essential [11,14]. Ultimately, this case underscores that management of laBCC with orbital invasion extends beyond achieving tumor response; it also requires addressing symptom burden, preserving functional outcomes, and aligning treatment strategies with patient goals through coordinated multidisciplinary care. Importantly, a favorable oncologic response does not necessarily equate to improvement in symptoms or quality of life. In such cases, referral to ocular oncology for consideration of palliative enucleation is warranted.

## Conclusions

laBCC with orbital invasion is an uncommon presentation associated with substantial morbidity. This case highlights the role of HHIs as neoadjuvant therapy in patients with periorbital BCC with orbital invasion. Although this patient demonstrated a favorable oncologic response to vismodegib, he continued to experience escalating pain and progressive vision loss, underscoring that tumor response does not necessarily translate into preservation of visual function or improvement in pain in advanced periorbital disease. Such patients may ultimately require surgical intervention to address persistent symptoms. In this case, palliative enucleation resulted in complete resolution of ocular pain, and the patient continued to show no evidence of disease recurrence four months after discontinuation of systemic cancer-directed therapy.

Coordinated multidisciplinary care is essential in the management of locally advanced periorbital BCC, where tumor control, functional outcomes, and symptom burden are closely interconnected. Prompt recognition of persistent or worsening symptoms and timely involvement of appropriate subspecialists, including ocular oncology and palliative care, are critical to achieving optimal patient-centered outcomes.
